# Common Mental Health Disorders and Their Current Prescription Patterns in Saudi Arabia's Primary Healthcare Settings

**DOI:** 10.7759/cureus.65562

**Published:** 2024-07-28

**Authors:** Jawza Alsabhan, Ashwaq Alanazi, Raghad Alhajaji, Malaz Elbashir, Faisal Alzahrani, Fatima Alhajaji, Mariah Almehmadi, Salihah Alqorashi, Bashaier Ahmed Fairaq, Fayza Alhazmi

**Affiliations:** 1 Clinical Pharmacy, King Saud University, Riyadh, SAU; 2 Second Health Cluster, King Fahad Medical City, Riyadh, SAU; 3 Public Health Department, Ministry of Health, Makkah, SAU; 4 Alhajj Primary Health Care, Primary Care Department, Makkah Health Cluster, Makkah, SAU; 5 Infectious Disease Department, Public Health Administration, Ministry of Health, Makkah, SAU; 6 School Health Department, Public Health Administration, Ministry of Health, Makkah, SAU; 7 College of Medicine, Umm Alqura University, Makkah, SAU; 8 Makkah Health Cluster, Health Promotion Administration, Makkah, SAU; 9 Family Medicine, Makkah Health Cluster, Makkah, SAU

**Keywords:** anxiety, primary health care centers, ssri, mental health, depression

## Abstract

Background

The mental healthcare program is widely implemented in primary healthcare settings in Saudi Arabia. It consisted of early screening and management of mental health disorders associated with chronic health conditions. Family physicians are authorized to prescribe selective serotonin reuptake inhibitors (SSRIs) for early management of mental health disorders in primary healthcare centers (PHCs). The aim of the study is exploring the prevalence of various types of mental health disorders and to assess the prescribing pattern of SSRIs in PHCs.

Materials and methods

A cross-sectional study based on the data from electronic health records, telephone interviews, and prescriptions of individuals administered SSRIs at PHCs.

Results

Among 219 patients visiting PHCs, 67.6% were female participants. Forty-four percent of the patients were 20-30 years old and 61.2% of them received SSRI medications. The most prevalent chronic condition was diabetes (22.5%) followed by hypertension (20.5%) and asthma (6.4%). The most prevalent mental disorder was major depressive disorder (MDD; 78.1%), followed by anxiety disorder (7.3%), panic disorder (6.4%), and MDD with anxiety disorders (5.5%).

Conclusion

Implementing mental healthcare programs in PHCs is believed to promote population health. MDD was the predominant mental health disorder among patients visiting PHCs, and SSRIs were the most prescribed medications in this setting. This suggests that mental healthcare programs in PHCs are effective in improving mental health outcomes.

## Introduction

Major depressive disorder (MDD), as defined by the Diagnostic and Statistical Manual of Mental Disorders, Fifth Edition (DSM-5), is a mental health condition characterized by the presence of one or more major depressive episodes [1]. To meet the criteria for MDD, an individual must experience five or more of the following symptoms during the same two-week period and represent a change from previous functioning; at least one of the symptoms must be either a depressed mood or loss of interest or pleasure [1]. A depressed mood is defined as feeling sad, empty, or hopeless most of the day, or nearly every day [1]. Loss of interest or pleasure is defined as markedly diminished interest or pleasure in all, or almost all, activities most of the day, nearly every day [1]. A significant weight change is defined as a noticeable increase or decrease in appetite or weight without intentional dieting [1]. Insomnia or hypersomnia means difficulty falling asleep, staying asleep, or sleeping excessively [1]. Psychomotor agitation, or retardation, is defined as observable restlessness or slowed movement and speech [1]. Fatigue, or energy loss, is defined as persistent feelings of tiredness or lack of energy [1]. Feelings of worthlessness or excessive guilt are defined as ongoing, unrealistic negative self-evaluations or feelings of guilt [1]. There is a reduction in the capacity to focus, make decisions, or experience a significant deterioration in cognitive abilities [1]. There are persistent thoughts of death, suicidal ideation, or an attempt at suicide [1].

According to the American Psychological Association's clinical practice guidelines for the treatment of depression, selective serotonin reuptake inhibitors (SSRIs) are increasingly utilized as the first-line treatment for MDD and other mental illnesses [1]. Additionally, SSRIs are preferred as they are generally safe and well-tolerated compared to other classes of antidepressants [2,3]. However, safety considerations and appropriate monitoring are still crucial for optimal patient outcomes [4]. Before SSRI treatment, to ensure the patient's health profile and avoid contraindication of the selected treatment, the required baseline assessments include information on medical conditions, allergies, previous medication use, a physical examination (e.g., vital signs, general health, and any existing physical health issues), and laboratory tests, including a complete blood count and liver function tests. Patients with heart conditions such as coronary artery disease and hypertension may benefit from electrocardiogram (ECG) monitoring to assess cardiac safety. ECG monitoring is also required for patients with high-risk factors for heart disease, such as smoking, obesity, and diabetes [5,6]. Regular monitoring for long-term SSRI use allows for dose adjustments and early detection of adverse effects [4].

Recent studies on SSRIs in Saudi Arabia indicate a growing interest in mental health therapy. Alhulwah et al. conducted a cross-sectional study that demonstrated a growing use of SSRIs over time, with 60% of SSRI prescriptions being in hospital settings [7]. This study identified the need for further investigation into prescription patterns as factors influencing optimal mental healthcare delivery. Al-Habeeb et al. investigated the prevalence of depression and anxiety disorders in primary care settings and highlighted the issues caused by these conditions on public health. These findings suggested that suitable SSRI prescription protocols are required to tackle increasing mental health concerns among the population [8,9].

In Saudi Arabia, mental healthcare services are integrated into primary healthcare centers, which family medicine consultants or registrars often oversee. These programs prioritize the early detection and treatment of prevalent mental health diseases such as MDD, generalized anxiety disorder, and panic disorder [10]. Family physicians who participated in this program provide drugs including SSRIs and tricyclic antidepressants for the initial treatment of mental health disorders [10]. Healthcare providers are instrumental in delivering timely and effective treatment and mental health support [10]. Mental health service patterns need more attention because healthcare providers should closely monitor drug interactions, side effects, and suicidal thoughts and behaviors. Effective monitoring and proactive management can help reduce risk and optimize treatment outcomes in individuals with depression [4,11-13].

Prescription practices must be assessed for the improvement of patient health and therapeutic efficacy and reduction of potential side effects associated with these medications. Therefore, this study's primary objective is to determine the prevalence of various mental health disorders among patients who visit primary healthcare centers in the Makkah region of Saudi Arabia. Secondary objectives include assessing SSRI prescribing patterns in these primary healthcare settings, identifying the demographic and clinical characteristics of patients receiving SSRIs, and investigating the relationship between SSRI use and chronic health conditions.

## Materials and methods

Study design and setting

Primary healthcare centers in the Makkah region conducted this cross-sectional study between April and October 2023.

Study population

**Figure 1:**
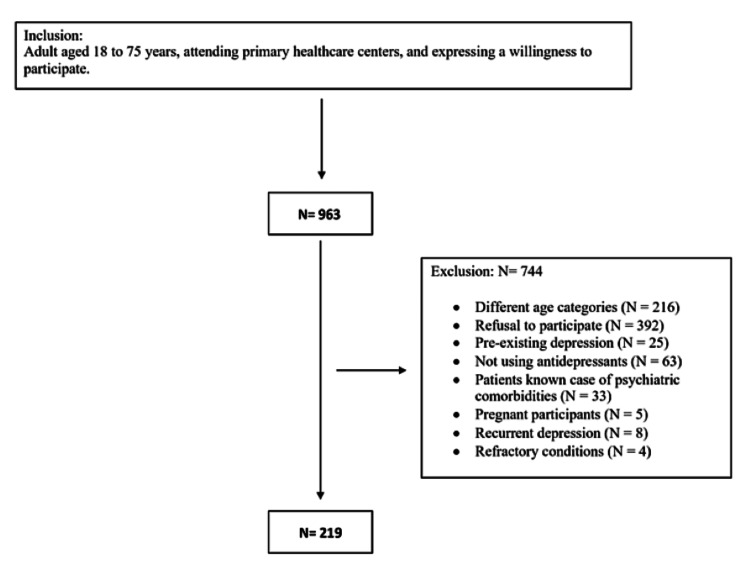
A flow diagram shows patients recruited in PHC settings in the Makkah region. PHC, primary healthcare.

We included participants, aged 18 to 75 years, seeking mental health services at primary healthcare centers. Exclusion criteria were individuals declining participation, those with pre-existing depression, non-users of antidepressants, individuals with psychiatric comorbidities, those requiring hospital referrals, pregnant participants, those with recurrent depression, and individuals with refractory conditions (Figure 1).

Sampling and recruitment

A pilot study determined the sample size to be approximately 219 participants, with a margin of error of 0.05 and a confidence level of 95%. We recruited participants using a simple random sampling technique from electronic health records (EHRs) who were enrolled in primary healthcare in the Makkah region seeking mental health services.

Data collection

Trained data collectors collected data from EHRs and conducted telephone interviews using a structured questionnaire to ensure consistency and accuracy. The EHR data included demographic information (age, gender, and BMI), chronic health conditions, and mental health diagnoses. A validated questionnaire was used for data acquisition, which was reviewed by three independent academic professors specializing in community medicine, and ensured reliability by conducting factor analysis.

Variables and measurements

The primary variables included demographic data, mental health diagnoses, chronic health conditions, and SSRI prescription patterns. Mental health disorders were diagnosed according to the Diagnostic and Statistical Manual of Mental Disorders, Fifth Edition (DSM-5) criteria and chronic conditions were reported based on the medical history and the EHRs.

Statistical analysis

Data were analyzed using IBM SPSS Statistics for Windows, version 27.0 (IBM Corp., Armonk, NY). We used descriptive statistics to summarize quantitative variables. Chi-square and Fisher's exact tests were applied based on variable types and distributions. Fisher’s exact test was utilized for small sample sizes to calculate the exact probability of observed frequencies, providing more accuracy than the chi-square test for small samples. A p-value less than 0.05 was considered statistically significant.

Ethical considerations

To maintain participant confidentiality, the research team anonymized the data, secured the electronic records, and used the data solely for research purposes. We obtained written informed consent from all participants, which included detailed information about the study's purpose, procedures, and potential risks. All participants had the right to refuse participation. Ethical approval was obtained from the Local Research Ethics Committee, Health of the Makkah Al-Mukarramah Region, and the Institutional Review Board (IRB), via Letter No. H-02-K-076-0223-896 dated March 26, 2023.

## Results

A total of 219 patients were seeking mental health services at primary healthcare centers in the Makkah region, and more than half of them, 134 (61.2%), were receiving SSRI medications. The majority of participants were female, 148 (67.6%). Most of the patients, 88 participants (44%), were within the age group of 20-30 years. Around 45.2% (n=99) of patients were diagnosed with chronic health conditions (Table 1).

**Table 1:** Demographic characteristics of the patients visiting the health primary care centers seeking mental health services. Significance level is considered at p <0.05.

Characteristics	No of participants, n (%)	Received SSRIs		Statistical test	Statistical test value	p-Value
		Yes, n (%)	No, n (%)			
Total no of participants, N (%)	219 (100)	134 (61.2)	83 (38.8)	Pearson chi-square		
Gender						
Male	71 (32.4)	39 (17.8)	32 (14.6)	Pearson chi-square	1.732	0.18
Female	148 (67.6)	95 (43.3)	53 (24.3)			
Age group						
20-30	88 (40.2)	54 (25)	34 (15.2)	Pearson chi-square	3.007	0.55
31-40	57 (26)	31 (14.2)	26 (11.8)			
41-50	39 (17.8)	28 (12.8)	11 (5)			
51-60	27 (12.3)	16 (7.3)	11 (5)			
>60	8 (3.7)	5 (2.2)	3 (1.5)			
Body mass index						
Underweight	6 (2.7)	2 (0.9)	4 (1.8)	Pearson chi-square	10.706	0.05
Normal	68 (31.1)	39 (17.8)	29 (17.1)			
Overweight	79 (36.1)	54 (0.9)	25 (5.8)			
Obese	38 (17.4)	23 (5.8)	15 (16.1)			
Extremely obese	24 (11)	16 (7.3)	8 (3.7)			
Missing	4 (1.8)	-	-			
Chronic health conditions						
Yes	99 (45.2)	57 (26)	42 (19.2)	Pearson chi-square	0.992	0.19
No	120 (54.8)	77 (35.1)	43 (19.7)			

Moreover, the results demonstrated that diabetes was the most frequent chronic condition diagnosed (n = 50, 22.5%). As a result, approximately 63% (n = 32) of those patients received SSRI medications. However, there was no significant association between the use of SSRIs and the incidence of diabetes (p = 0.64). Patients diagnosed with hypertension (n = 45, 20.5%) and asthma (n = 14, 6.4%) (p = 0.36, 0.74), respectively, also showed this association. Patients suffering from thyroid disease reached a significant level (p = 0.01) (Table 2).

**Table 2:** Prevalence of chronic conditions in patients visiting the primary healthcare centers seeking mental health services. *Significance level is considered at p <0.05.

Chronic conditions	Prevalence, n (%)	Received SSRIs, n (%)	Statistical test	Statistical test value	p-Value
Diabetes	50 (22.5)	32 (64)	Fisher's exact test	0.216	0.64
Hypertension	45 (20.5)	30 (66)		6.407	0.36
Hypothyroidism	18 (8.2)	6 (33)		0.101	0.01*
Asthma	14 (6.4)	8 (57.1)		0.103	0.74
Other conditions	29 (13.2)	16 (5.5)		0.509	0.47

Table 3 presents the current mental health complaints reported by the participants with the highest percentage of sleep disorder (n = 120, 54.8%), followed by feeling tired (n = 103, 37%) and poor appetite or overeating (n = 99, 45.2%). In comparison, the least common complaint was thought or attempted suicide (n = 24, 11%). None of them reached a significant level.

**Table 3:** The current complaints mentioned by participants who received mental health services at the primary healthcare centers.

Complaint	Frequency (%)	Received mental health services (N)		Test	Statistical test value	p-Value
		Yes	No			
Little interest in hobbies	70 (32)	42	28	Fisher's exact test	0.061	0.85
Feeling tired	103 (47)	65	38		0.302	0.58
Sleep disorder	120 (54.8)	68	52		2.284	0.13
Poor appetite or overeating	99 (45.2)	63	36		0.456	0.29
Thought or attempt to suicide	24 (11)	13	11		0.683	0.71
Mood disorder	95 (43.4)	53	42		2.058	0.09

The study's findings show that the primary psychiatric diagnosis, MDD, was the predominant diagnosis (n = 171, 78.1%), followed by anxiety disorder (n = 16, 7.3%), panic disorder (n = 14, 6.4%), and MDD with anxiety disorders (n = 12, 5.5%) (Table 4).

**Table 4:** The prevalence of psychiatric disorders among who received mental health services at the primary healthcare centers.

Diagnosis	Frequency	Percent
Major depression disorder	171	78.1
Anxiety	16	7.3
Panic disorder	14	6.4
Major depressive disorder and anxiety disorders	12	5.5

## Discussion

The study's findings present a comprehensive analysis of the psychiatric diagnoses and medication prescribing among patients seeking mental health services at primary healthcare centers. In 2011, a prior study in the southeastern region of Saudi Arabia estimated a 12% incidence of depression among patients [12]. Recent observational research conducted in the Al-Ahsa region in Saudi Arabia elucidated that one-third of attendees at primary healthcare centers experienced depression [13]. Despite the COVID-19 pandemic, a study indicated that depression rates did not witness a significant increase [14]. However, the pandemic identified elevated rates of anxiety, likely due to social deprivation and the absence of rehabilitation support [14].

Our findings indicate that diabetes is the most prevalent chronic illness in our population, with 22.5% (n = 55) and 64% (n = 32) of them receiving SSRI medication, respectively. Recent research has the link between antidepressant use and glycemic control, and it showed that the use of multiple antidepressant subclasses in adults with diabetes significantly raised HbA1C levels, posing a potential risk for suboptimal glycemic control [15-17]. Diabetes itself tends to increase the risk of MDD [16]; while short-term antidepressant treatment benefits insulin sensitivity and mitigates depression in non-diabetic patients, long-term effects may show an adverse effect [15,16]. Prolonged use of antidepressants strongly correlates with an increased risk of developing diabetes, which indicates that it is the antidepressant medication itself, rather than the condition of depression, that may play a role in the development of type 2 diabetes [16,17]. According to our study, SSRI drugs are the widely prescribed medication for depression and anxiety in Saudi Arabia's primary healthcare centers; escitalopram in particular is the most common SSRI prescribed, and it is considered a well-tolerated and safe medication with a low side-effect profile [5]. In primary healthcare settings in Canada, escitalopram can be used with confidence to treat patients with MDD [11]. However, SSRIs need careful initiation and monitoring parameters, especially in patients with comorbid conditions, pregnancy or lactation, and the elderly [11,18]. For instance, the use of SSRIs may well be associated with night sweats in older patients in primary care settings [11]. Furthermore, gastrointestinal distress with escitalopram can be managed by using escitalopram oral drops instead of oral tablets [19].

One of the major strengths of this study is its focus on primary healthcare centers, providing real-world insights into the psychiatric diagnoses and prescription patterns in a primary care setting. The use of EHRs and telephone interviews allowed for comprehensive data collection, ensuring that various aspects of patient health and treatment were captured. Additionally, the study's focus on SSRIs, particularly escitalopram, highlights the practical application of these medications in a real-world primary care setting, contributing valuable information to existing literature. This study, like other studies, has several limitations. The small sample size and cross-sectional design may have limited the ability to fully capture the effectiveness and safety outcomes of SSRIs, given the number of study variables and the data collection timeframe. Moreover, social desirability bias occurs when people make more positive comments in order to influence how others perceive them. It includes the practice of self-deceptive enhancement, which involves providing positively biased self-reports, as well as impression management [20]. This social desirability may have impacted the one-on-one interview methodology with each patient. Furthermore, the study did not account for potential confounding factors such as socioeconomic status, lifestyle factors, and the presence of other comorbid conditions that might influence the study findings. Also, some of the non-significant correlations could be due to small frequencies in categorical variables, which could explain some of the negative results due to a lower statistical power. Therefore, larger-scale, multicenter studies are warranted to validate the findings in this current study.

## Conclusions

Screening for mental health disorders in primary care settings is an essential program for improving population health and quality of life. SSRIs are the most commonly given antidepressant drugs in Saudi Arabia's primary care settings due to their high safety and tolerability profile. Furthermore, primary healthcare clinics must have an experienced mental health practitioner on hand to provide guidance and advice. All primary care physicians were recommended to receive regular training to ensure they were aware of the hazards associated with SSRIs.
